# Late presentation of iatrogenic dissection of right coronary cusp: A case report

**DOI:** 10.34172/jcvtr.2020.55

**Published:** 2020-12-02

**Authors:** Ali Eshraghi, Majid Jalalyazdi, Javad Ramezani, Mostafa Baburian

**Affiliations:** ^1^Department of Cardiovascular Diseases, Faculty of Medicine, Mashhad University of Medical Sciences, Mashhad, Iran

**Keywords:** Coronary Angiography, Iatrogenic, Coronary Artery, Dissection

## Abstract

Iatrogenic dissection of coronary arteries while performing catheter engagement, in general is not uncommon. However, we encountered a relatively rare case of iatrogenic right coronary cusp dissection.Here we report an iatrogenic coronary artery dissection after diagnostic angiography in a 54-year-oldwoman presented with exertional dyspnea and chest discomfort. In our case delayed progression of sub-intimal hematoma and subsequent compression of RCA ostium an SA node branch was the cause of SA node dysfunction and subsequent junctional rhythm and atrial fibrillation.

To conclude it should be said that in catastrophic cases of iatrogenic coronary ostia dissection and ensuing aortic cusp involvement, stenting of entry point at coronary ostia is a logical decision with good result.

## Introduction

In recent years, trans-radial artery approach has become popular among cardiologists for both diagnostic coronary angiography and percutaneous coronary intervention (PCI). This is mostly due to its superiority to traditional femoral approach, which provides less discomfort for the patient and the ability to walk immediately after procedure, less bleeding, shorter hospital stay and even the possibility of performing the procedure in out-patient settings.^1^

Iatrogenic coronary artery dissection following the angiography of coronary arteries is a rare condition with high mortality rate if not diagnosed and treated early.^2^ It is reported that its incidence is as low as 0.02%. However, it should be mentioned that the rate of iatrogenic coronary artery dissection after PCI is almost twenty times higher compared to its incidence after coronary angiography.^3^

There are several factors contributing to development of iatrogenic coronary artery dissection including: mal alignment of diagnostic or guiding catheter with coronary ostia, the structure of the guiding wire, coronary arteries’ anatomy and the operator factors (experience and technical skill).^1^

In this report we present a case of catheter-induced (iatrogenic) right coronary sinus and ascending aortic dissection following diagnostic angiography.

## Case Presentation

A 54-year-old woman with diabetes was referred to the cardiology clinic with dyspnea and chest discomfort on moderate exertion. ECG and Physical examination was normal. Transthoracic echocardiogram showed normal ejection fraction, grade 1 diastolic dysfunction, no significant valvular dysfunction and no pathology in other chamber and pericardium. Because of the moderate pretest probability of coronary artery disease (CAD), exercise treadmill test (ETT) was done which was positive for CAD. The patient was scheduled for coronary angiography via right radial artery.

**Figure 1:**
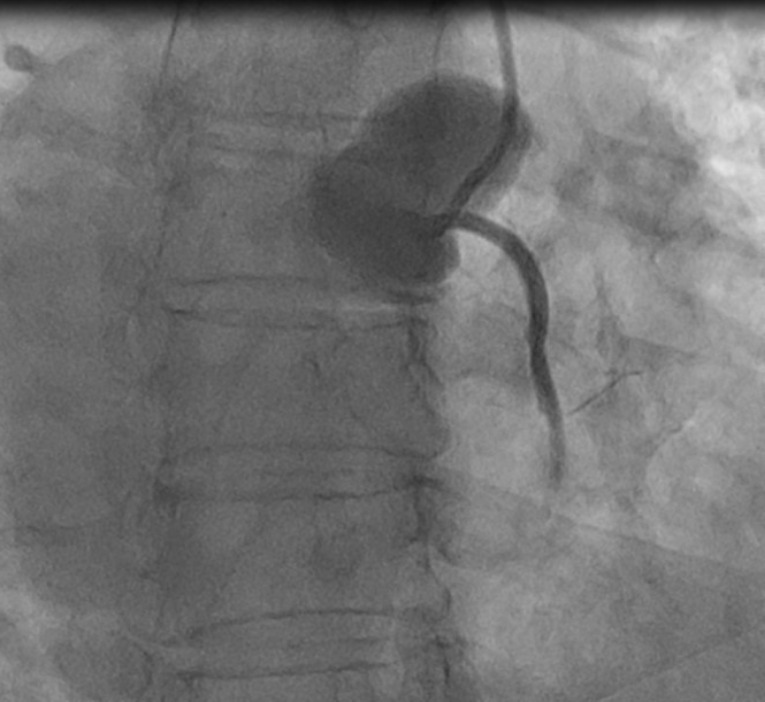
Angiography of Right Coronary Artery

After the first dye injection in the right coronary artery (RCA), a radiopaque area was seen in the right coronary sinus that expanded to the ascending aorta, and patient experienced chest pain, suggesting catheter-induced (iatrogenic) right coronary sinus and ascending aortic dissection (Figure 1).

Right coronary artery also showed no coronary disease and the patient had stable condition. A few minutes later patient’s chest pain decreased and ECG did not show any sign of ischemia. Bed side echocardiography (TTE) was then performed to evaluate for a dissection flap. By TTE, no abnormality was noted. Computed tomography (CT) also demonstrated no evidence of aortic dissection and sub intimal hematoma.

Since the patient’s condition was stable, it was decided to perform watch and wait strategy and therefore she was treated non-surgically, with aggressive blood pressure control.

**Figure 2:**
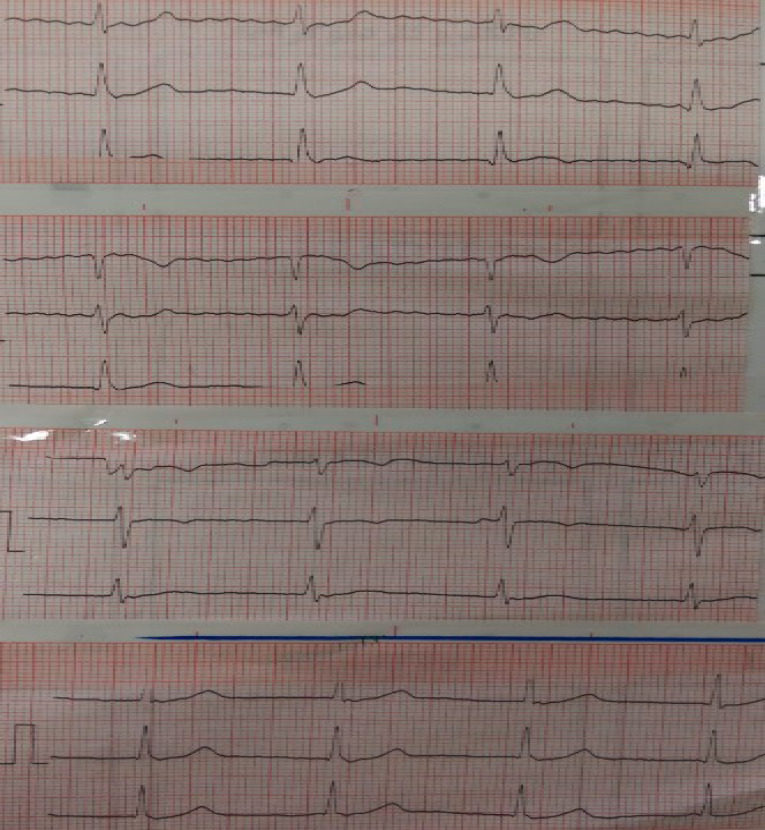
Eight hours after initial treatment: ECG showing junctional rhythm with no ischemic change

**Figure 3:**
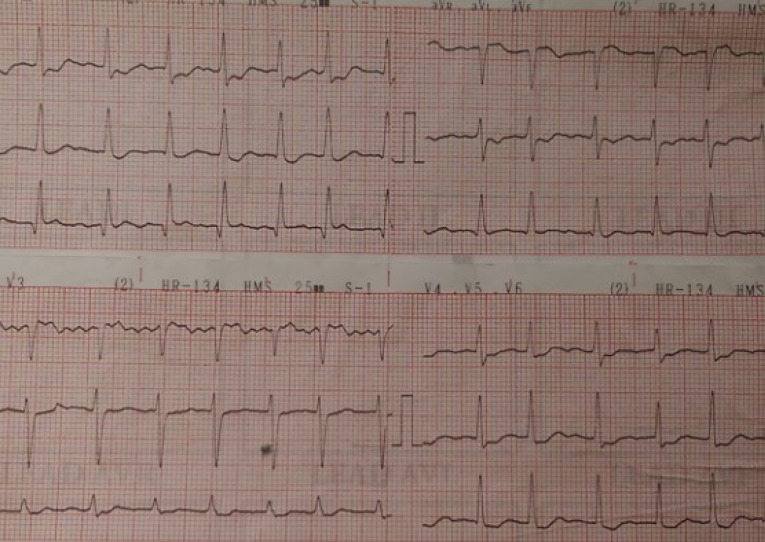
Fourteen hours after initial treatment: Atrial Fibrilation occurred

After 8 hours the patient experienced mild chest discomfort. ECG showed junctional rhythm with no ischemic change (Figure 2). Six hours later her blood pressure decreased and atrial fibrillation was observed in ECG (Figure 3).

Echocardiography was repeated and showed normal EF. No pericardial effusion but there was a mass compressing RCA ostium.

**Figure 4:**
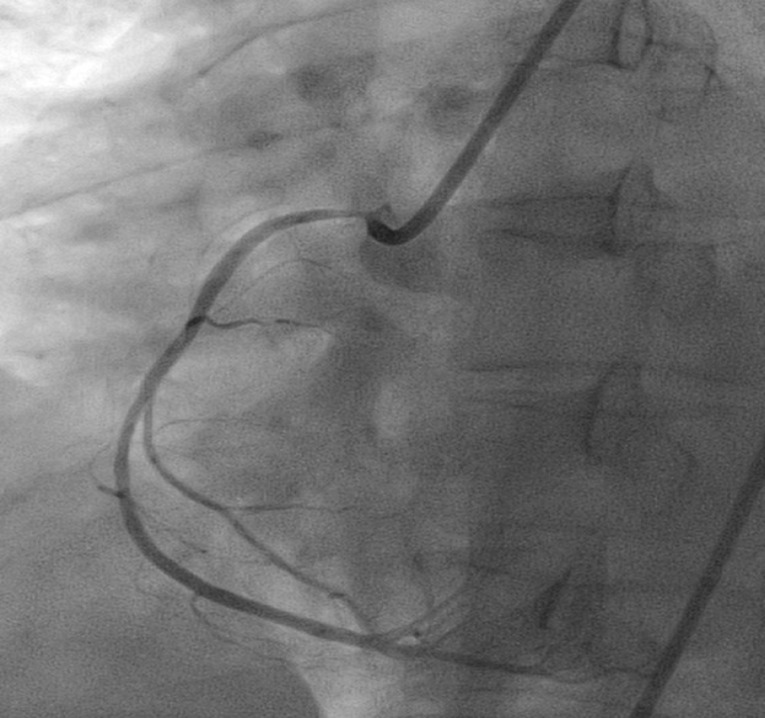
Reapeated Coronary Angiography

We decided to repeat the coronary angiography. Angiography was performed via right femoral artery. LM, LAD and LCX was normal. Right coronary injection showed severe narrowing and slit like RCA ostium .SA node artery was absent (Figure 4).

**Figure 5:**
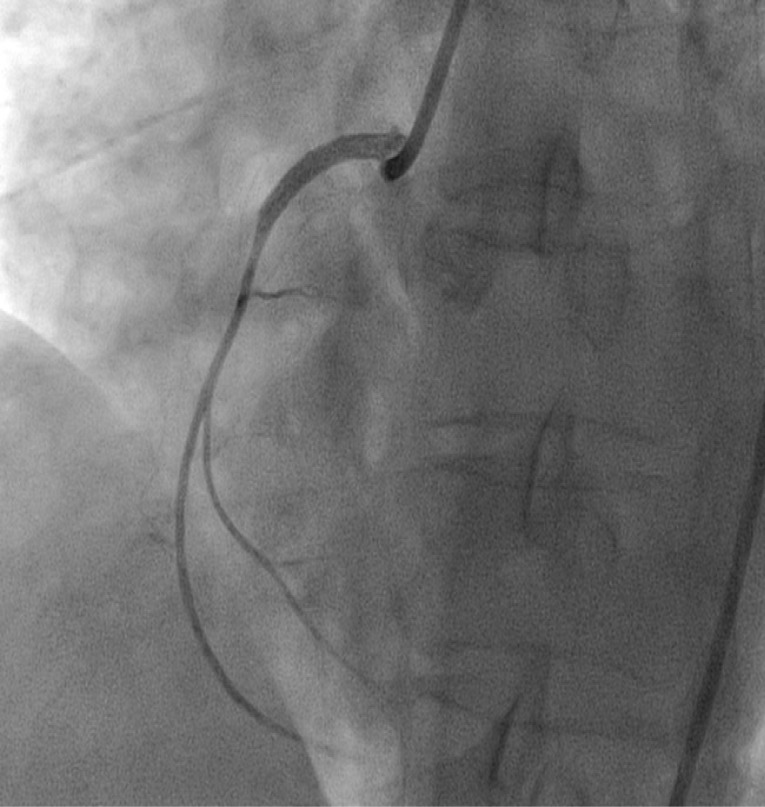
Final Coronary Angiography

DES stent 3*28 was deployed from RCA-ostium to proximal RCA. Subsequent injection showed no further contrast staining of the aortic wall. The flow in the RCA was normalized (Figure 5).

Two hours after PCI of RCA, ECG was normal sinus rhythm and the patient had no chest pain. Follow-up echocardiography revealed normal left ventricular function and a normal aortic root.

## Discussion

Catheter induced aortic dissection has a reported incidence of only 0.02%.^4^ Coronary artery dissection may result in complete occlusion of the true vessel lumen by compression from the false lumen.^5^ It represents a potentially lethal complication. Both surgery and PCI of the coronary ostia to seal the entry site have been reported as treatment options.^4^

There are several mechanisms responsible for this complication. It appears that catheter trauma first produces coronary artery dissection which can then progress retrogradely through the subintima into the aortic root. Vigorous hand injection of contrast medium, subintimal passage of the guidewire, and improper manipulation of the catheter have been proposed as possible mechanisms of iatrogenic coronary dissection.^6^

In our case we observed that we thought that catheter mal alignment resulted in coronary ostial dissection and further injection of the dye into the subintimal space was the cause of the retrograde progression of the dissection. Forceful contrast injections should be avoided since it promotes propagation of flap dissection. Susceptibility to the development of intimal tears and progression of a hematoma may be related to underlying structural weakness of the media.^4^ The predisposing factors include hypertension, congenital unicuspid and bicuspid aortic valves, Marfan syndrome, cystic media necrosis and extensive atherosclerosis.^4,7^ In one original study, the incidence of proximal RCA dissection increased with selection of a Kimny®, Amplatz or EBU guiding catheter, atherosclerotic plaque lesions in the proximal RCA, angulation (> 90°) of the proximal RCA and various degrees of calcification of the proximal RCA.^1^

If the vessel flow is compromised the most important point is rapid restoration of vessel patency. In previous reports, surgery, PCI and conservative management have all been successful.^8-10^ CABG is best suited for left main (LM) artery lesions, multi-vessel disease ^11^. PCI is an alternative treatment for this condition. In an original study^9^ and a case report,^12^ bail-out stenting for treating catheter-induced LM dissection was technically feasible and showed good short- and long-term results. In a case series by Leontyev and colleagues^13^ with dissections generally progressing into the aorta, surgical management was performed in all cases. By contrast, in a recently published series by Nunez and colleagues,^14^ the patients were treated conservatively, being followed up by close monitoring with imaging. They concluded conservative strategy and stenting of coronary ostia when dissected is a logical approach with good short- and long-term prognosis.

In 2002, Dunning et al published a series of 9 patients with coronary dissection extending to the aorta (incidence, 0.02%) and proposed a classification in 3 grades: type 1, dissection limited to the sinuses of Valsalva; type 2, dissection of the ascending aorta outside of the sinuses < 4 cm; and type 3, dissection ≥ 4 cm. These authors proposed that stent implantation was sufficient in the limited forms, but those with type 3 required surgery.^4^

## Conclusion

In catastrophic cases of iatrogenic coronary ostia dissection and ensuing aortic cusp involvement, stenting of entry point at coronary ostia is a logical decision with good result.
